# MobileSteelNet: A Lightweight Steel Surface Defect Classification Network with Cross-Interactive Efficient Multi-Scale Attention

**DOI:** 10.3390/s26031022

**Published:** 2026-02-04

**Authors:** Xiang Zou, Zhongming Liu, Chengjun Xu, Jiawei Zhang, Zhaoyu Li

**Affiliations:** Jiangxi Provincial Key Laboratory of Intelligent Information Processing and Affective Computing, School of Artificial Intelligence, Jiangxi Normal University, Nanchang 330022, China; 202226703019@jxnu.edu.cn (Z.L.); 005627@jxnu.edu.cn (C.X.); zhangjiawei@jxnu.edu.cn (J.Z.); 202341600139@jxnu.edu.cn (Z.L.)

**Keywords:** steel surface defect classification, lightweight neural network, multi-scale feature fusion, cross-interactive attention

## Abstract

Steel surface defect classification is critical for industrial quality control, yet existing methods struggle to balance accuracy and efficiency for real-time deployment in vision-based sensor systems. This paper presents MobileSteelNet, a lightweight deep learning framework that introduces two novel modules: multi-scale feature fusion (MSFF), for integrating multi-stage features; and Cross-Interactive Efficient Multi-Scale Attention (CIEMA), which unifies inter-channel interaction, parallel multi-scale spatial extraction, and grouped efficient computation. Experiments on the NEU-DET dataset demonstrate that MobileSteelNet achieves 91.36% average accuracy, surpassing ResNet-50 (88.01%) and lightweight networks, including MobileNetV2 (86.08%). Notably, it achieves 93.70% accuracy on Scratch-type defects, representing an 82.12 percentage point improvement over baseline MobileNetV1. With a model size of only 8.2 MB, MobileSteelNet maintains superior performance while meeting lightweight deployment requirements, making it suitable for edge deployment in vision sensor systems for steel manufacturing.

## 1. Introduction

**Figure 1:**
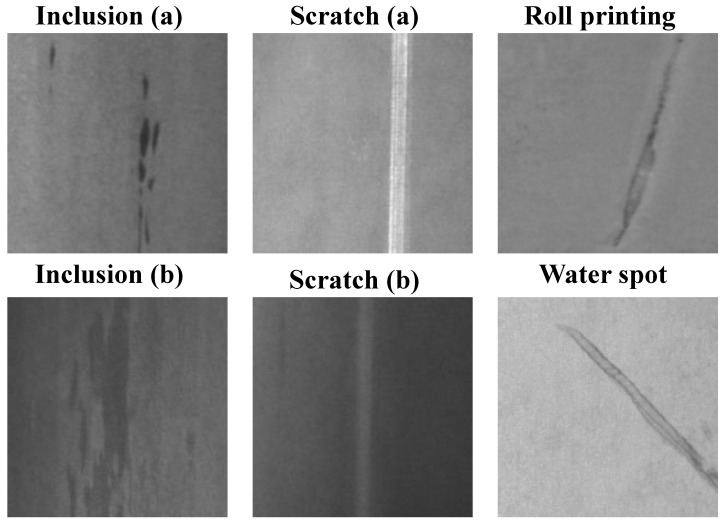
Steel surface defect exemplars: (1) large-scale variation—Inclusion (a) remains tiny while Inclusion (b) expands to a much larger region; (2) diverse imaging conditions—Scratch (a) is captured under good illumination with high contrast, whereas Scratch (b) suffers from poor lighting and low contrast; and (3) high inter-class similarity—roll printing and water spot defects exhibit similar appearances and are prone to confusion.

Steel, as one of the fundamental materials in modern manufacturing, plays a crucial role in numerous industrial fields, such as construction, automotive, and infrastructure development [1]. Six common types of surface defects in steel production—namely Crazing, Inclusion, Patches, Pitted Surface, Rolled-in Scale, and Scratches—each exhibit unique visual characteristics and formation mechanisms [2,3]. These defects shorten steel life and cost millions, making automated visual inspection a manufacturing must [4]. As illustrated in Figure 1, steel surface defect detection still faces multiple challenges in practical applications. First, the size and morphology of defects vary drastically, among which tiny defects may only occupy a few pixels and are highly likely to be missed during the feature extraction stage. Second, the imaging conditions in the production line fluctuate sharply; factors such as uneven illumination, specular reflection, or mechanical vibration will introduce a large amount of noise, resulting in completely different visual characteristics of the same type of defect under different scenarios. Third, there is a high degree of visual similarity among multiple defect categories, which often leads to misjudgment by classification models. However, manual inspection is labor intensive, fatigue prone, and subjective, compromising reliability, cost, and consistency. Therefore, developing automated, accurate, and efficient vision-based sensor systems for steel surface defect detection has become an urgent need in the industry, driving the widespread application of computer vision and deep learning technologies in this field [5,6,7].

Early automated defect detection adopted traditional machine learning techniques that relied on manually crafted geometric, shape, and texture features, yielding limited generalization [8]. Under complex backgrounds and varying illumination, the heavy feature-engineering burden and volatile performance fell short of industrial demands [9]. To address the aforementioned limitations, the rise of deep learning techniques—especially the advent of convolutional neural networks (CNNs)—has brought revolutionary breakthroughs to this field through their end-to-end automatic feature extraction and hierarchical representation learning capabilities [10]. Deep network architectures, such as ResNet [11], VGG [12], and Inception, have demonstrated excellent performance in multiple defect detection benchmark tests. For instance, methods based on the improved ResNet-50 have achieved high detection accuracy on real-world factory environment datasets [3], and transfer learning methods using VGG16 as the feature extractor have also exhibited outstanding performance [13]. However, such models usually contain a large number of parameters and have high computational complexity, making them difficult to deploy on edge devices in industrial real-time scenarios [3].

Consequently, this limitation has prompted the development of lightweight neural networks. The MobileNet series [14,15] employs depthwise separable convolution (DSConv) to decompose standard convolution into depthwise and pointwise convolution steps, reducing computational cost and parameter count by approximately 8–9 times compared to traditional convolution. However, lightweight models still have room for improvement in feature representation capability when handling subtle defects and limited samples [16].

In parallel, attention mechanisms have emerged as powerful tools for enhancing feature representation, improving model performance by selectively emphasizing information-rich regions or channels. Representative studies on the channel attention mechanism include the Squeeze-and-Excitation Network (SE-Net) [17]. This model generates channel weights via global average pooling and two fully connected layers, which can improve the performance of ResNet-50 on the ImageNet classification task with only a slight increase in the number of parameters. Efficient Channel Attention (ECA) [18] replaces fully connected layers with one-dimensional convolution, avoiding dimension reduction and further reducing computational overhead while maintaining performance.

In addition to channel attention, the spatial attention mechanism focuses on the importance differences among different positions in feature maps and generates spatial weight maps through pooling and convolution operations [19]. The Convolutional Block Attention Module (CBAM) [19] sequentially processes channel and spatial dimension information in a cascaded manner, further improving the recognition accuracy of ResNet-50 on the ImageNet dataset. Coordinate attention [20] integrates position information encoding into the channel attention mechanism; models the spatial dependencies in horizontal and vertical directions, respectively; and achieves significant performance improvements on mobile networks, such as MobileNetV2. Efficient Multi-Scale Attention (EMA) [21] maintains lightweight characteristics, and it aggregates information from different receptive fields through a multi-scale branch structure to realize efficient multi-scale feature modeling.

Despite the remarkable progress achieved in the aforementioned research, the application of deep learning techniques to the practical industrial scenarios of steel defect detection still faces the following core challenges. First, the contradiction between accuracy and efficiency has not been effectively resolved. Second, existing attention mechanisms suffer from a design flaw of insufficient cross-interaction among multi-scale feature channels. These limitations indicate that there is an urgent need for a novel technical solution that organically integrates lightweight architecture design, efficient attention mechanisms, and multi-scale feature fusion to achieve a balance between high detection accuracy and engineering deployability.

To address the aforementioned challenges, this paper proposes a lightweight steel surface defect classification network with Cross-Interactive Efficient Multi-Scale Attention, namely MobileSteelNet. Based on MobileNetV1, this network adds two innovative modules—multi-scale feature fusion (MSFF) and Cross-Interactive Efficient Multi-Scale Attention (CIEMA)—which collaboratively enhance the model’s feature fusion capability.

The main contributions are summarized as follows:

(1) This paper proposes a Cross-Interactive Efficient Multi-Scale Attention (CIEMA) mechanism. This mechanism integrates channel interaction, coordinate encoding, and cross-spatial learning to simultaneously capture local fine-grained details and global semantic dependencies; it also adaptively balances channel and spatial information via a dynamic gating mechanism, thereby enhancing the model’s discriminative ability for similar defects.
(2) A multi-scale feature fusion (MSFF) module was designed to aggregate hierarchical features from three distinct stages of the backbone network, and it bridges the gap between shallow textural details and deep semantic representations, significantly improving the model’s robustness against drastic defect scale variations.
(3) A lightweight network named MobileSteelNet was constructed, which achieves an average precision of 91.36% on the NEU-DET dataset, providing a solution that balances high accuracy and high efficiency for edge deployment in industrial scenarios.

The remainder of this paper is organized as follows. Section 2 presents the MobileSteelNet architecture, detailing the MSFF and CIEMA module designs. Section 3 describes the experimental setup and reports comprehensive results, including comparisons with baseline methods and ablation studies. Section 4 discusses the findings, limitations, and future work. Section 5 concludes the paper. Code is available at https://github.com/DWlzm/MobileSteelNet (accessed on 25 January 2026).

## 2. Methods

### 2.1. Overall Framework

**Figure 2:**
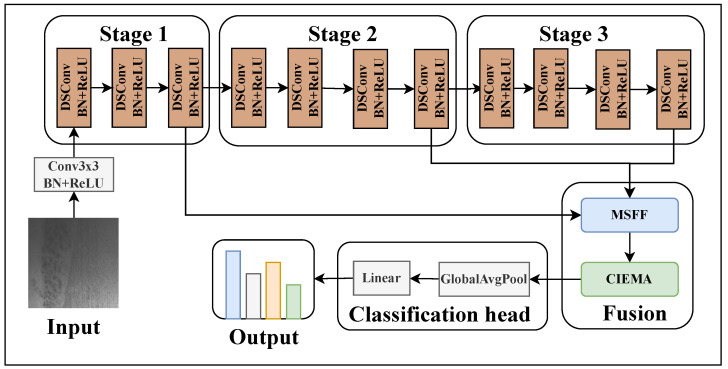
Schematic diagram of MobileSteelNet network structure.

The overall framework is shown in Figure 2, and the specific tensor transformations are shown in Table 1. MobileSteelNet maintains the efficient structure of MobileNetV1 while effectively enhancing the model’s feature representation capability through attention enhancement and multi-scale fusion mechanisms.

Specifically, the input image is $X_{0}\in R^{C\times H\times W}$, where C=3 represents the number of RGB channels, and H,W represent the height and width of the input image. Moreover, $X_{0}$ first enters the Init Layer, where the number of channels is expanded from 3 to 32 through 32 convolution kernels of size 3×3. Since these 32 convolution kernels have a stride of 2 and the feature map undergoes zero padding (padding = 1), the width and height of the output feature map are reduced to half of the original image, resulting in output $X_{1}\in R^{C_{1}\times \frac{H}{2}\times \frac{W}{2}}$, where $C_{1}=32$. Subsequently, to extract feature maps with rich semantic information, $X_{1}$ needs to pass through three consecutive feature extraction stages, namely Stage 1, Stage 2, and Stage 3.

Stage 1 contains three DSConv (depthwise separable convolution) modules for progressively extracting features and increasing the number of channels. Consecutive DSConv blocks are repeated to increase representational capacity and receptive field while keeping parameters and FLOPs low; this enables the network to capture both fine-grained textures and higher-level context efficiently. First, the input feature map $X_{1}$ passes through DSConv, increasing the number of channels from $C_{1}=32$ to $C_{2}=64$, outputting the feature map $X_{2}\in R^{C_{2}\times \frac{H}{2}\times \frac{W}{2}}$. Then, $X_{2}$ passes through DSConv with stride 2, reducing the spatial size of the feature map by half and increasing the number of channels to $C_{3}=128$, resulting in the output feature map $X_{3}\in R^{C_{3}\times \frac{H}{4}\times \frac{W}{4}}$. Finally, feature map $X_{3}$ passes through a DSConv, resulting in the output feature map $X_{4}\in R^{C_{3}\times \frac{H}{4}\times \frac{W}{4}}$.

Stage 2 contains four DSConv modules for further feature extraction and channel number increase. The input feature map $X_{4}$ passes through DSConv, increasing the number of channels from $C_{3}=128$ to $C_{4}=256$, while maintaining the spatial size of the feature map unchanged, i.e., the output feature map $X_{5}\in R^{C_{4}\times \frac{H}{4}\times \frac{W}{4}}$. Next, $X_{5}$ passes through a DSConv operation with stride 2, reducing the spatial size of the feature map by half and increasing the number of channels to $C_{5}=512$, outputting the feature map $X_{6}\in R^{C_{5}\times \frac{H}{8}\times \frac{W}{8}}$. In the third and fourth modules of Stage2, feature map $X_{6}$ passes through two DSConv operations, with the number of channels remaining unchanged, outputting the feature maps $X_{7}\in R^{C_{5}\times \frac{H}{8}\times \frac{W}{8}}$ and $X_{8}\in R^{C_{5}\times \frac{H}{8}\times \frac{W}{8}}$, respectively.

Stage 3 contains four DSConv modules for extracting high-level features. The first two DSConv operations perform DSConv on the input feature map $X_{8}$, with the number of channels remaining unchanged, outputting the feature maps $X_{9}$ and $X_{10}$ (both with dimensions $R^{C_{5}\times \frac{H}{8}\times \frac{W}{8}}$). The third module uses DSConv with stride 2, reducing the spatial size of the feature map by half and increasing the number of channels to $C_{6}=1024$, outputting the feature map $X_{11}\in R^{C_{6}\times \frac{H}{16}\times \frac{W}{16}}$. The fourth DSConv maintains the number of channels for $X_{11}$, outputting the feature map $X_{12}\in R^{C_{6}\times \frac{H}{16}\times \frac{W}{16}}$.

After completing three-stage feature extraction, to further enhance feature representation capability, a multi-scale feature fusion (MSFF) module is introduced. The MSFF module fuses the output feature maps $X_{4}$, $X_{8}$, and $X_{12}$ from Stage 1, Stage 2, and Stage 3. Specifically, the MSFF module first uniformly scales all input feature maps to the spatial resolution of the largest input feature map (i.e., $\frac{H}{4}\times \frac{W}{4}$) through bilinear interpolation, then concatenates them along the channel dimension, and finally obtains the fused feature map $X_{13}\in R^{C_{6}\times \frac{H}{4}\times \frac{W}{4}}$ through 1×1 convolution, batch normalization, and ReLU activation, where $C_{6}=1024$. This multi-scale feature fusion mechanism enables the model to comprehensively capture fine-grained to coarse-grained feature information, accurately identifying both smaller-scale Inclusion and larger-scale Patches, thereby significantly enhancing feature robustness and representation capability.

Based on multi-scale feature fusion, the model further enhances channel interaction through CIEMA (Cross-Interactive Efficient Multi-Scale Attention). The CIEMA module processes the fused feature map $X_{13}$, generating the enhanced feature map $X_{14}\in R^{C_{6}\times \frac{H}{4}\times \frac{W}{4}}$. Through channel interaction enhancement, the model can more effectively utilize information between different channels, further improving feature representation capability.

Finally, the model converts the feature map $X_{14}$ into a global feature vector $X_{15}\in R^{C_{6}}$ through global average pooling (GAP). GAP compresses the spatial dimension to 1×1 by calculating the average value of each channel. Subsequently, $X_{15}$ is fed into a fully connected layer, generating the final output $\hat{Y}=WX_{15}+b$, where *W* is the weight matrix and *b* is the bias vector. The fully connected layer maps the global feature vector to the target category space, outputting the model’s prediction results.

In summary, through this design, MobileSteelNet significantly enhances the model’s feature representation capability by introducing attention enhancement and multi-scale feature fusion mechanisms while maintaining the efficient structure of MobileNetV1, thereby achieving superior performance in steel surface defect detection tasks.

### 2.2. Multi-Scale Feature Fusion Module

**Figure 3:**
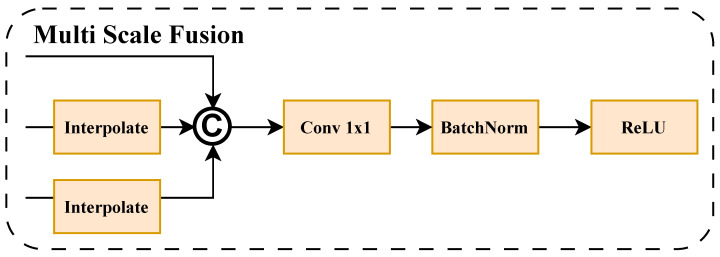
Structural diagram of the multi-scale feature fusion module.

As mentioned earlier, feature maps from different stages contain semantic information at different scales. To effectively fuse and integrate feature map information from different receptive fields, a multi-scale feature fusion (MSFF) module was designed. As shown in Figure 3, the MSFF module fuses the output feature maps $X_{4}$, $X_{8}$, and $X_{12}$ from Stage1, Stage2, and Stage3, generating the fused feature map $X_{13}\in R^{C_{6}\times \frac{H}{4}\times \frac{W}{4}}$, where $C_{6}=1024$. For convenience in describing the generic module, the inputs of MSFF are denoted as follows: $\{F_{1}\in R^{C_{1}\times H_{1}\times W_{1}},\;F_{2}\in R^{C_{2}\times H_{2}\times W_{2}},\;F_{3}\in R^{C_{3}\times H_{3}\times W_{3}}\}.$ The processing procedure of the MSFF module is divided into three steps. First, to achieve cross-scale information alignment, the MSFF module uniformly scales all feature maps to the spatial resolution $\left(H_{\max},W_{\max}\right)$ of the largest input feature map through bilinear interpolation:

(1) $$F_{i}=\mathrm{Interp}\left(F_{i},\;\left(H_{\max},W_{\max}\right)\right),H_{\max}=\max\left(H_{1},H_{2},H_{3}\right),\;W_{\max}=\max\left(W_{1},W_{2},W_{3}\right).$$

Subsequently, the adjusted features are concatenated along the channel dimension to obtain aggregated features:

(2) $$F_{\mathrm{cat}}=\mathrm{Concat}\left(F_{1},\;F_{2},\;F_{3}\right)\in R^{\left(C_{1}+C_{2}+C_{3}\right)\times H_{\max}\times W_{\max}}.$$

Finally, to further achieve channel compression and information interaction, the module employs a fusion layer consisting of 1×1 convolution, batch normalization, and the ReLU activation function:

(3) $$F_{\mathrm{out}}=\mathrm{ReLU}\left(\mathrm{BN}\left({\mathrm{Conv}}_{1\times 1}\left(F_{\mathrm{cat}}\right)\right)\right)\in R^{C_{\mathrm{out}}\times H_{\max}\times W_{\max}},$$

where $C_{\mathrm{out}}$ is the number of output channels, which can be set according to task requirements.

This design achieves efficient cross-scale information aggregation while keeping computational overhead controllable, providing richer contextual representations for subsequent attention enhancement and classification prediction.

### 2.3. Cross-Interactive Efficient Multi-Scale Attention

**Figure 4:**
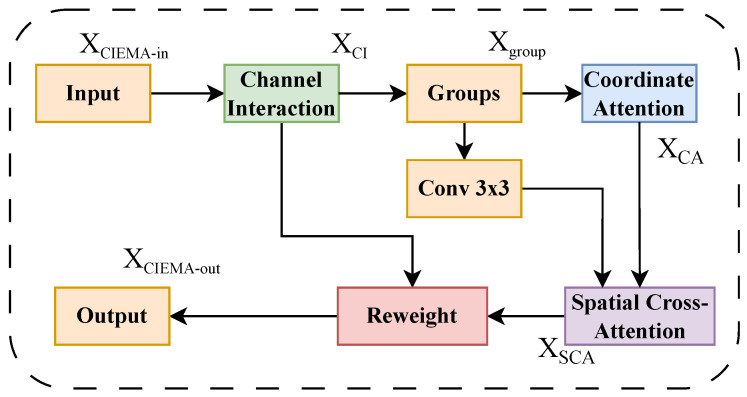
Structural diagram of Cross-Interactive Efficient Multi-Scale Attention.

After completing multi-scale feature fusion, to further enhance feature representation capability while maintaining computational efficiency, a novel attention module—Cross-Interactive Efficient Multi-Scale Attention (CIEMA) —is proposed. As shown in Figure 4, this module mainly consists of two core components: (1) Channel interaction, for modeling global channel dependencies; and (2) Cross-Spatial Learning, for characterizing fine-grained spatial structures under grouped features.

Specifically, the feature map $X_{13}$ adjusted by MSFF serves as the input to the CIEMA module, which is denoted as $X_{\mathrm{CIEMA}-\mathrm{in}}$. The processing flow of the CIEMA module is as follows.

Step 1: Channel dependency modeling. First, dependency relationships are established for each channel of the feature map $X_{\mathrm{CIEMA}-\mathrm{in}}$ through the channel interaction module, resulting in feature map $X_{\mathrm{CI}}$.

Step 2: Grouped processing and coordinate attention. After establishing channel dependencies, to reduce computational complexity, grouped operations are applied to $X_{\mathrm{CI}}$, resulting in $X_{\mathrm{group}}=\left\{X_{\mathrm{group}}^{\left(1\right)},X_{\mathrm{group}}^{\left(2\right)},\ldots ,X_{\mathrm{group}}^{\left(G\right)}\right\}$, where G≪C. Subsequently, the grouped features are fed into coordinate attention to achieve spatial focusing, resulting in $X_{\mathrm{CA}}=\left\{X_{\mathrm{CA}}^{\left(1\right)},X_{\mathrm{CA}}^{\left(2\right)},\ldots ,X_{\mathrm{CA}}^{\left(G\right)}\right\}$.

Step 3: Local and global information fusion. Since $X_{\mathrm{CA}}$ uses 1×H and W×1 pooling to obtain global spatial semantic information but lacks local semantic information, the grouped features are fed into the Cross Spatial Learning (CSL) module to obtain multi-scale descriptors $X_{\mathrm{CSL}-\mathrm{out}}=\left\{X_{CSL-out}^{\left(1\right)},X_{CSL-out}^{\left(2\right)},\ldots ,X_{CSL-out}^{\left(G\right)}\right\}$, which aggregate fine-grained local semantics.

Step 4: Spatial cross-attention fusion. The feature map $X_{\mathrm{CA}}$ with global contextual semantic information and the CSL output $X_{\mathrm{CSL}-\mathrm{out}}$ with local semantic information are fed into the Spatial Cross Attention module to fuse global semantic information and local information, resulting in feature map $X_{\mathrm{SCA}}$.

Step 5: Residual connection and gated fusion. Finally, to avoid losing channel dependency relationships after adjustment by the Spatial Cross Attention module, feature map recalibration is achieved through residual connection and dynamic gating mechanism, resulting in the output $X_{\mathrm{CIEMA}-\mathrm{out}}$ of the CIEMA module.

The design details of each submodule are described in depth below.

#### 2.3.1. Channel Interaction

**Figure 5:**
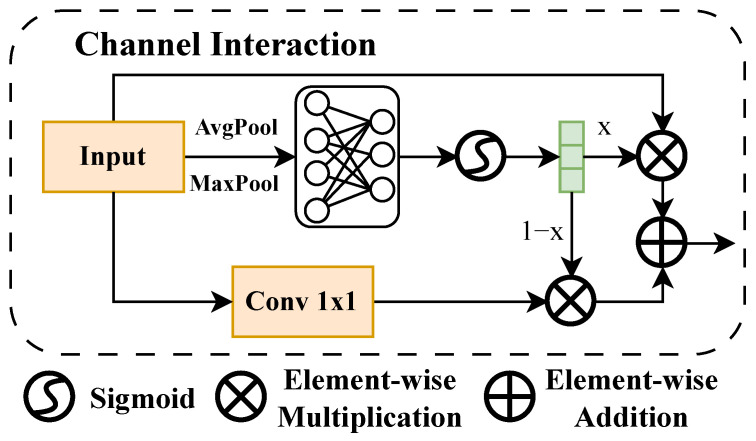
Structural diagram of channel interaction.

Unlike some grouped attention methods, CIEMA first performs interaction modeling within the full channel range to ensure capturing complete channel dependencies. As shown in Figure 5, let the input feature map be $X_{\mathrm{CIEMA}-\mathrm{in}}\in R^{B\times C\times H\times W}$. Additionally, the channel statistics are obtained through global average pooling and max pooling:

(4) $$z_{\mathrm{avg}}=\mathrm{GAP}\left(X_{\mathrm{CIEMA}-\mathrm{in}}\right),\;z_{\max}=\mathrm{GMP}\left(X_{\mathrm{CIEMA}-\mathrm{in}}\right).$$

Nonlinear channel dependencies are extracted through a shared MLP, and then channel attention weights are obtained through Sigmoid activation:

(5) $$A_{c}=\mathrm{Sigmoid}\left(f\left(z_{\mathrm{avg}}\right)+f\left(z_{\max}\right)\right).$$

Meanwhile, to explicitly model inter-channel interaction relationships, 1×1 convolution is employed:

(6) $$X_{\mathrm{int}}={\mathrm{Conv}}_{1\times 1}\left(X_{\mathrm{CIEMA}-\mathrm{in}}\right).$$

Channel-enhanced features are then, finally, obtained through weighted fusion:

(7) $$X_{\mathrm{CI}}=X_{\mathrm{CIEMA}-\mathrm{in}}⊗A_{c}+X_{\mathrm{int}}⊗\left(1-A_{c}\right).$$

#### 2.3.2. Coordinate Attention

**Figure 6:**
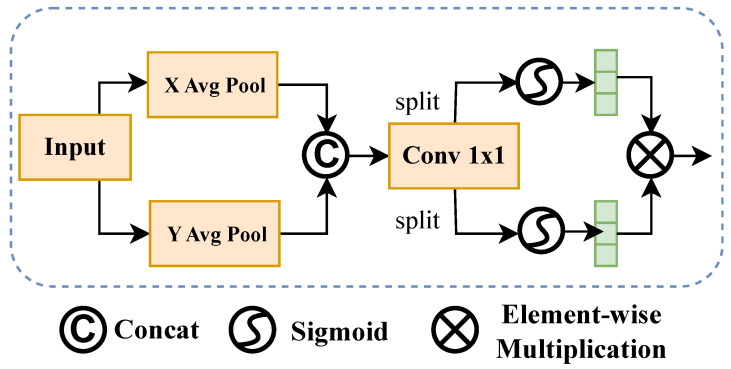
Structure of the coordinate attention module.

After inter-channel interaction, CIEMA further introduces the Coordinate Attention mechanism to fully capture the spatial position information and directional dependencies of features. Unlike traditional global attention, coordinate attention aggregates spatial information along horizontal and vertical directions separately, thereby obtaining more precise positional information representation while maintaining lightweight characteristics. The structure diagram of coordinate attention is shown in Figure 6.

First, the channel-enhanced features $X_{c}\in R^{B\times C\times H\times W}$ output from the previous stage are divided according to the number of groups *G*, with each group containing C/G channels:

(8) $$X_{c}\in R^{B\times C\times H\times W}\;\;⟶\;\;\left\{X_{c}^{\left(1\right)},X_{c}^{\left(2\right)},\ldots ,X_{c}^{\left(G\right)}\right\}.$$

Then, for each group of input feature maps, global average pooling is performed along two spatial directions separately to obtain direction-sensitive descriptors:

(9) $$Z_{h}\left(c,i\right)=\frac{1}{W}\sum_{j=1}^{W}X_{c}^{\left(i\right)}\left(c,i,j\right),\;Z_{w}\left(c,j\right)=\frac{1}{H}\sum_{i=1}^{H}X_{c}^{\left(i\right)}\left(c,i,j\right),$$

where $Z_{h}\in R^{B\times C\times H\times 1}$ represents the aggregation result along the horizontal direction, and $Z_{w}\in R^{B\times C\times 1\times W}$ represents the aggregation result along the vertical direction.

Subsequently, the two are concatenated and then fused through a shared convolution layer and nonlinear activation function ReLU:

(10) $$f=\mathrm{ReLU}\left({\mathrm{Conv}}_{1\times 1}\left([Z_{h},Z_{w}]\right)\right).$$

Next, the fused features are mapped back to the attention weight space of horizontal and vertical directions through two independent 1×1 convolution layers:

(11) $$A_{h}=\mathrm{Sigmoid}\left({\mathrm{Conv}}_{1\times 1}^{h}\left(f\right)\right),\;A_{w}=\mathrm{Sigmoid}\left({\mathrm{Conv}}_{1\times 1}^{w}\left(f\right)\right).$$

Finally, the two directional attentions are reapplied to the input features to achieve adaptive enhancement of the spatial dimension:

(12) $$X_{\mathrm{CA}}=X_{c}⊗A_{h}⊗A_{w}.$$

Through this process, CIEMA not only preserves global dependencies between channels, but also introduces direction-sensitive spatial information, enabling the model to more precisely locate key regions and feature patterns, providing more discriminative representations for subsequent feature fusion and semantic extraction.

#### 2.3.3. Spatial Cross Attention

**Figure 7:**
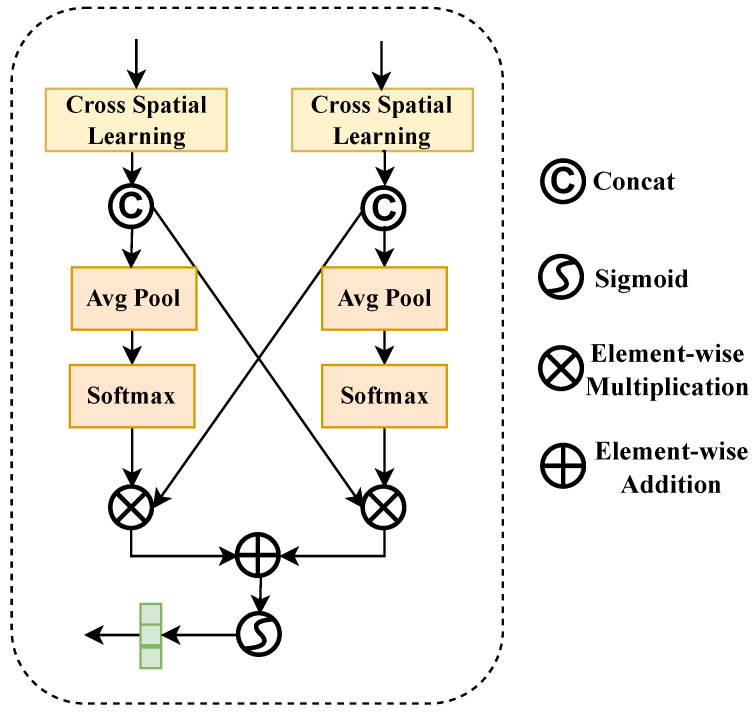
Structural diagram of Spatial Cross Attention.

After obtaining the coordinate attention-enhanced features $X_{\mathrm{CA}}$ and local features $X_{3\times 3}$, the Spatial Cross Attention module is responsible for effectively fusing the two. As shown in Figure 7, the feature map $X_{\mathrm{CA}}=\left\{X_{\mathrm{CA}}^{\left(1\right)},X_{\mathrm{CA}}^{\left(2\right)},\ldots ,X_{\mathrm{CA}}^{\left(G\right)}\right\}$ with global contextual semantic information and the feature map $X_{3\times 3}=\left\{X_{3\times 3}^{\left(1\right)},X_{3\times 3}^{\left(2\right)},\ldots ,X_{3\times 3}^{\left(G\right)}\right\}$ with local semantic information are fed into the Spatial Cross Attention module to fuse global semantic information and local information, resulting in feature map $X_{\mathrm{SCA}}$.

The Spatial Cross Attention module first performs Cross Spatial Learning on the two input information to obtain multi-scale contextual information $X_{\mathrm{CSL}-\mathrm{out}}$ to better capture defect features at different scales.

Subsequently, $X_{\mathrm{CSL}-\mathrm{out}}$ is concatenated to obtain feature map $X_{\mathrm{CSL}-\mathrm{out}}\prime$:

(13) $$X_{\mathrm{CSL}-\mathrm{out}}^{\prime }=\left[X_{\mathrm{CSL}-\mathrm{out}}^{\left(1\right)},X_{\mathrm{CSL}-\mathrm{out}}^{\left(2\right)},\ldots ,X_{\mathrm{CSL}-\mathrm{out}}^{\left(G\right)}\right].$$

Finally, $X_{\mathrm{CSL}-\mathrm{out}}^{\prime }$ is subjected to global average pooling to obtain global descriptor V, and then a weight vector $V^{\prime }$ is generated through Softmax:

(14) $$\begin{array}{c} V=\mathrm{GAP}\left(X_{\mathrm{CSL}-\mathrm{out}}^{\prime }\right),\;V^{\prime }=\mathrm{Softmax}\left(V\right). \end{array}$$

#### 2.3.4. Cross Spatial Learning Module

**Figure 8:**
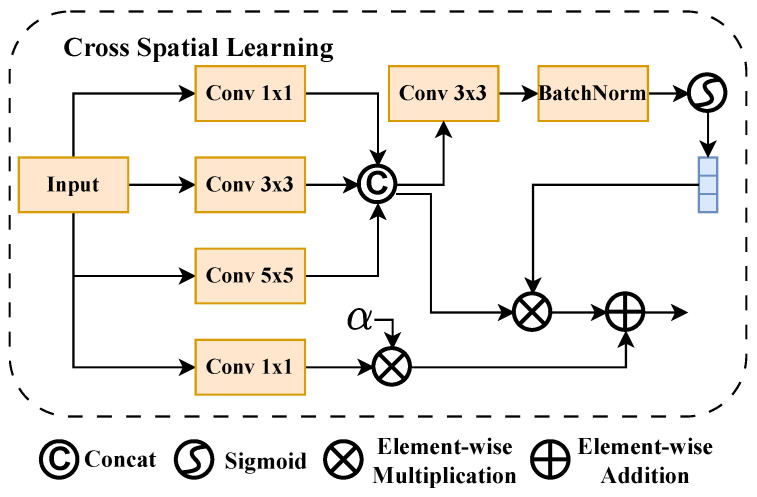
Structural diagram of Cross Spatial Learning.

As shown in Figure 8, to extract multi-scale spatial information from the input feature map $X_{\mathrm{CSL}-\mathrm{in}}$, it is processed through convolution kernels of different sizes. These different convolution branches can capture spatial structures at different levels in the feature map, thereby obtaining multi-scale spatial representations.

Specifically, three different-sized convolution operations are first applied to the input feature map $X_{\mathrm{CSL}-\mathrm{in}}$ to obtain three scale feature maps:

(15) $$\begin{array}{cccc} X_{1\times 1} & ={\mathrm{Conv}}_{1\times 1}\left(X_{\mathrm{CSL}-\mathrm{in}}\right), & X_{3\times 3} & ={\mathrm{Conv}}_{3\times 3}\left(X_{\mathrm{CSL}-\mathrm{in}}\right),\\ X_{5\times 5} & ={\mathrm{Conv}}_{5\times 5}\left(X_{\mathrm{CSL}-\mathrm{in}}\right), & X_{\mathrm{fused}} & =[X_{1\times 1},X_{3\times 3},X_{5\times 5}], \end{array}$$

where $X_{1\times 1}$, $X_{3\times 3}$, and $X_{5\times 5}$ are feature maps obtained through 1×1, 3×3, and 5×5 convolutions, respectively; and $X_{\mathrm{fused}}$ is the fused feature map obtained by concatenating them along the channel dimension.

Next, a 1×1 convolution layer is applied to the concatenated fused feature map $X_{\mathrm{fused}}$, combined with batch normalization and Sigmoid activation function, to generate a spatial attention map $A_{s}$. This spatial attention map can weight features at different positions in the spatial dimension, highlighting features in important regions while suppressing unimportant parts:

(16) $$A_{s}=\mathrm{Sigmoid}\left({\mathrm{BatchNorm}(\mathrm{Conv}}_{1\times 1}\left(X_{\mathrm{fused}}\right)\right)),\;X_{\mathrm{fused}}^{\prime }=A_{s}⊗X_{\mathrm{fused}}.$$

Moreover, to further model dependency relationships between the different channels of the feature map $X_{\mathrm{CSL}-\mathrm{in}}$, a 1×1 convolution branch is introduced. This branch obtains channel weight representation $X_{\mathrm{local}}$ through convolution operations to capture inter-channel relationships:

(17) $$X_{\mathrm{local}}={\mathrm{Conv}}_{1\times 1}\left(X_{\mathrm{CSL}-\mathrm{in}}\right).$$

Finally, the multi-scale feature map $X_{\mathrm{fused}}^{\prime }\in R^{H\times W\times 3C}$ is reduced to dimension $X_{\mathrm{fused}}^{\prime \prime }\in R^{H\times W\times C}$ and fused with the channel dependency feature map $X_{\mathrm{local}}$ through weighted fusion to obtain the final output $X_{\mathrm{CSL}-\mathrm{out}}$:

(18) $$X_{\mathrm{CSL}-\mathrm{out}}=X_{\mathrm{fused}}^{\prime }+X_{\mathrm{local}}⊗\alpha ,$$

where α is a learnable parameter that adjusts the contribution weight of $X_{\mathrm{local}}$ through data-driven learning.

Through this approach, cross-spatial learning can fully exploit spatial information and inter-channel dependencies in the input feature map, thereby enhancing the diversity and richness of feature representations.

#### 2.3.5. Spatial Cross-Attention Fusion

After processing through the Cross Spatial Learning module, the two inputs $\{X_{3\times 3}^{\left(1\right)},$ $X_{3\times 3}^{\left(2\right)},\ldots ,X_{3\times 3}^{\left(G\right)}\}$ and $\left\{X_{\mathrm{CA}}^{\left(1\right)},X_{\mathrm{CA}}^{\left(2\right)},\ldots ,X_{\mathrm{CA}}^{\left(G\right)}\right\}$ are concatenated separately:

(19) $$I_{1}=\left[\left\{X_{3\times 3}^{\left(1\right)},X_{3\times 3}^{\left(2\right)},\ldots ,X_{3\times 3}^{\left(G\right)}\right\}\right],\;I_{2}=\left[\left\{X_{\mathrm{CA}}^{\left(1\right)},X_{\mathrm{CA}}^{\left(2\right)},\ldots ,X_{\mathrm{CA}}^{\left(G\right)}\right\}\right],$$

where $I_{1}$ is the local semantic information feature map, and $I_{2}$ is the global semantic information feature map with positional information obtained through coordinate attention. Subsequently, global average pooling is performed on $I_{1},I_{2}$ to obtain channel feature descriptors:

(20) $$I_{\mathrm{GAP}1}=\mathrm{GAP}\left(I_{1}\right),\;I_{\mathrm{GAP}2}=\mathrm{GAP}\left(I_{2}\right).$$

Then, importance weights are generated for each channel through a Softmax function:

(21) $$W_{1}=\mathrm{Softmax}\left(I_{\mathrm{GAP}1}\right),\;W_{2}=\mathrm{Softmax}\left(I_{\mathrm{GAP}2}\right),$$

where $W_{1}$ and $W_{2}$ represent weight vectors calculated through the Softmax function, which are used to measure the importance of different channel features.

Finally, cross-fusion of local features $I_{1}$ and global features $I_{2}$ is achieved to better aggregate contextual information and extract key features:

(22) $$F_{\mathrm{SCA}-\mathrm{out}}=W_{1}⊗I_{2}+W_{2}⊗I_{1}.$$

#### 2.3.6. Final Weighted Fusion

After completing Spatial Cross Attention processing, to further model inter-channel dependencies of $F_{\mathrm{SCA}-\mathrm{out}}$, the Squeeze-and-Excitation (SE) approach is employed for feature recalibration.

First, a 1×1 convolution layer is used to reduce the number of channels from *C* to $\frac{C}{4}$, and the nonlinear representation capability of features is enhanced through batch normalization (BN) and ReLU activation function, resulting in intermediate features $F_{\mathrm{multi}-\mathrm{scale}}^{\prime }$. Subsequently, another 1×1 convolution layer is used to restore the number of channels to the original dimension *C*, resulting in the recalibrated feature map $F_{\mathrm{multi}-\mathrm{scale}}^{\prime \prime }$:

(23) $$\begin{array}{c} F_{\mathrm{multi}-\mathrm{scale}}^{\prime }=\mathrm{ReLU}\left(\mathrm{BN}\left({\mathrm{Conv}}_{1\times 1,C\to \frac{C}{4}}\left(F_{\mathrm{SCA}-\mathrm{out}}\right)\right)\right),\\ F_{\mathrm{multi}-\mathrm{scale}}^{\prime \prime }=\mathrm{BN}\left({\mathrm{Conv}}_{1\times 1,\frac{C}{4}\to C}\left(F_{\mathrm{multi}-\mathrm{scale}}^{\prime }\right)\right). \end{array}$$

Then, the space-enhanced feature $F_{\mathrm{SCA}-\mathrm{out}}$ and multi-scale feature $F_{\mathrm{multi}-\mathrm{scale}}^{\prime \prime }$ are concatenated along the channel dimension, and gate weights are generated through the final fusion layer (1×1 convolution, batch normalization, and Sigmoid activation):

(24) $$\begin{array}{c} F_{\mathrm{combined}}=\mathrm{Concat}\left(F_{\mathrm{SCA}-\mathrm{out}},F_{\mathrm{multi}-\mathrm{scale}}^{\prime \prime }\right)\\ A_{p}=\mathrm{Sigmoid}\left(\mathrm{BN}\left({\mathrm{Conv}}_{1\times 1}\left(F_{\mathrm{combined}}\right)\right)\right). \end{array}$$

Finally, to avoid losing channel dependency relationships after processing through coordinate attention and Spatial Cross Attention, CIEMA achieves fusion of $X_{\mathrm{CI}}$ (channel interaction-enhanced features) and $F_{\mathrm{SCA}-\mathrm{out}}$ (spatial-enhanced features) through a dynamic gating mechanism to complete final feature adjustment:

(25) $$F_{\mathrm{CIEMA}-\mathrm{out}}=X_{\mathrm{CI}}⊗A_{p}+F_{\mathrm{SCA}-\mathrm{out}}⊗\left(1-A_{p}\right).$$

Through this gated fusion mechanism, the CIEMA module can adaptively balance channel interaction information and spatial cross-attention information, ensuring that the final output features simultaneously possess global channel dependency and fine-grained spatial perception capability.

## 3. Experiment

### 3.1. Dataset and Experimental Setup

#### 3.1.1. Dataset Description

**Figure 9:**
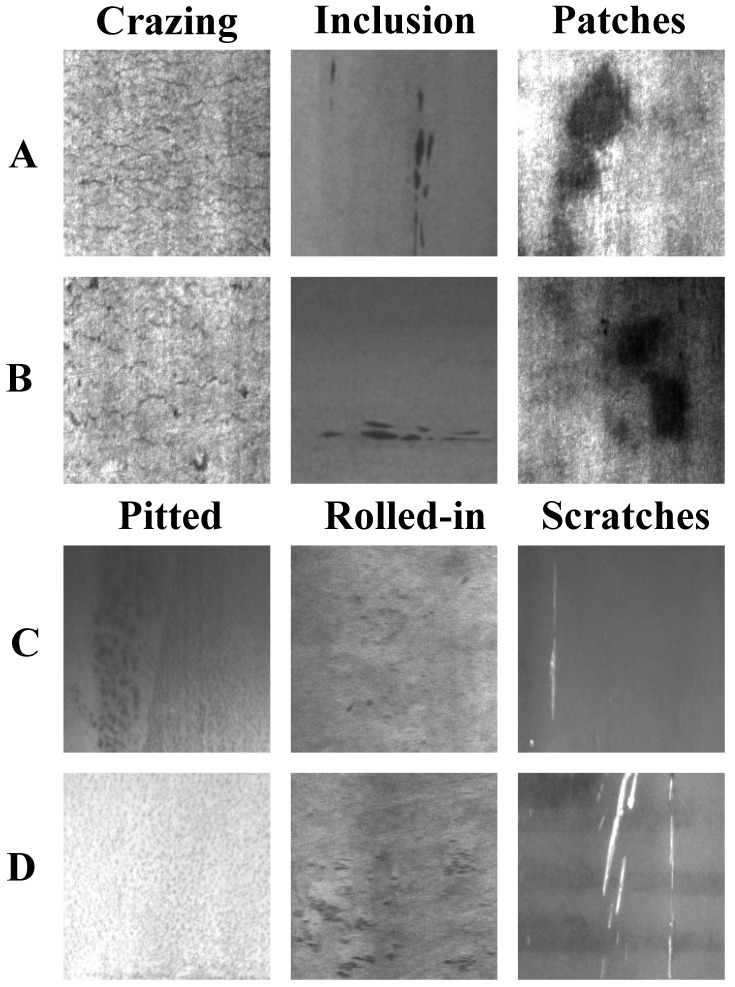
Sample images (**A**–**D**) from the NEU-DET dataset.

Experiments were conducted on the NEU-DET dataset. The NEU-DET dataset was created by Song Kechen’s team at Northeastern University, focusing on detection and recognition of steel surface defects. The dataset contains 1800 images covering six common types of steel surface defects: Crazing, Inclusion, Patches, Pitted Surface, Rolled-in Scale, and Scratches. Figure 9 illustrates sample images from the NEU-DET dataset, and Table 2 details the characteristics of various defects.

#### 3.1.2. Dataset Contrast Analysis

To deeply analyze the visual feature differences of steel surface defects, contrast statistical analysis was conducted on six defect categories in the NEU-DET dataset. Contrast is defined as the standard deviation of the image, representing the brightness differences in the image, and it is commonly used to measure image detail and texture clarity. Table 3 presents the contrast statistics for each category, including the maximum, minimum, and median values.

From the statistical data, it can be observed that there are significant differences in contrast among different defect categories. Specifically, the Patches category has the highest contrast (maximum value of 74.21), indicating that detail and texture variations in its images are relatively pronounced and can be detected more readily. In contrast, the Inclusion category has a minimum contrast of only 5.97, meaning that the brightness differences in defect regions of its images are small, which may impede the distinction of defects from the background. Furthermore, Crazing and Scratches have contrast values at moderate levels (51.54 and 49.02, respectively), showing that these two types of defects have certain visual details, but their contrast is still lower than the Patches category, which may pose higher challenges to the accuracy of detection algorithms.

**Figure 10:**
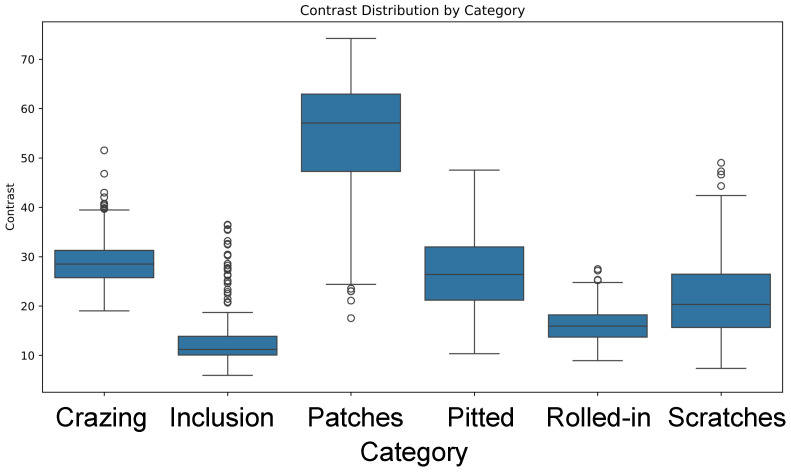
Contrast box plot of NEU-DET.

This contrast distribution difference indicates that detecting these categories requires high generalization capability and fine-grained feature extraction from algorithms. Consequently, this observation serves as one of the important motivations for the design of multi-scale feature fusion and attention mechanisms. The box plot in Figure 10 visually illustrates the distribution of contrast for each category.

#### 3.1.3. Experimental Configuration

To evaluate the performance of different models on the steel surface defect classification task, the following experimental configuration was adopted. Regarding the optimizer, the experiments used the Adam optimizer with an initial learning rate of 0.001, and the learning rate was dynamically adjusted during training through warmup learning rate scheduling. We adopted grid search to determine the optimal hyper-parameters for each algorithm on the NEU-DET dataset. For each hyperparameter combination, we performed 5-fold cross-validation on the validation set to ensure robust performance estimation. All experiments were conducted on a computer equipped with an NVIDIA GeForce RTX 3090 graphics card (NVIDIA Corporation, Santa Clara, CA, USA) (24 GB memory). The lightweight design of MobileSteelNet (8.2 MB) makes it suitable for deployment on edge devices and embedded vision sensor systems commonly used in industrial inspection. For the data split, we deliberately adopted an extreme ratio of 5% for training, 5% for validation, and 90% for testing to simulate the “data scarcity” scenario in industrial settings, and this was performed so as to verify the generalization ability of the proposed method with extremely limited labeled samples. We set the upper limit of training epochs to 200, and the training process was terminated early if the validation loss did not decrease for 10 consecutive epochs; the training/validation loss and average accuracy were recorded in each epoch. We set the random seed to 42 and fixed the split of the training and test sets of the dataset, ensuring that the relative performance of MobileSteelNet remained consistent. When only 5% of the training data was utilized, we adopted five lightweight data-augmentation strategies to alleviate overfitting and to enhance the model’s generalization ability: random horizontal flip with probability 0.5 to enforce directional invariance; random rotation within $\pm 15^{\circ }$ to simulate viewpoint deviations; color jitter that perturbs brightness, contrast, and saturation by ±10% and hue by ±5% for robustness to illumination and color shifts; and random affine transformation with translation ±5% and scaling in [0.9,1.1] to improve adaptability to position and scale variations. All baseline models were trained from scratch without the use of pre-trained weights. The final parameters were selected based on highest average validation accuracy across all folds, with secondary consideration for training stability. The selected values (learning rate = 0.001, batch size = 32, weight decay = 1 $\times \;10^{-4}$, and patience = 10) represent the optimal balance between convergence speed and generalization for lightweight CNNs on the steel defect classification task.

In terms of loss function, the Cross-Entropy Loss was employed to measure the difference between model predictions and true labels. The cross-entropy loss function is commonly used for multi-classification tasks, and it is defined as follows:

(26) $$L=-\sum_{i=1}^{N}y_{i}\log\left({\hat{y}}_{i}\right),$$

where *N* is the number of categories, $y_{i}$ is the true label of the sample, and ${\hat{y}}_{i}$ is the probability value predicted by the model. For a sample, the loss function measures the gap between the model’s predicted probability and the true label: the smaller the value, the more accurate the model’s prediction.

### 3.2. Comparative Experiments

#### 3.2.1. Comparison with Mainstream Network Architectures

To comprehensively evaluate the performance of different deep neural networks on steel-surface defect classification, we conducted systematic comparative experiments on the NEU-DET dataset. The evaluated models span the heavyweight ResNet family (ResNet18, ResNet34, and ResNet50) [11]; lightweight MobileNet family (MobileNetV1 [14], MobileNetV2 [15], and MobileNetV3 [22]); the efficient ShuffleNet family (ShuffleNetV1 [23] and ShuffleNetV2 [24]); and the latest efficient backbones MobileOne-S0 [25], EdgeNeXt-S [26], RepViT-M1 [27], and FastViT-SA12 [28]. Table 4 reports the detailed classification results of all the compared models.

From the experimental results, several key observations can be made: (1) among the heavy networks, ResNet50 achieved the best performance with an average accuracy of 88.01%, but it had high parameter count and computational complexity; (2) among the lightweight networks, the original MobileNetV1 exhibited weak performance (70.29%), especially achieving only 11.58% recognition rate on the Scratches category; and (3) the proposed MobileSteelNet achieved the highest average accuracy of 91.36% while maintaining lightweight characteristics—its 8.2 MB footprint is substantially smaller than heavy baselines—surpassing all comparative models. Notably, it achieved a recognition rate of 93.70% on the Scratches category, representing an improvement of 82.12 percentage points compared to the baseline MobileNetV1.

**Figure 11:**
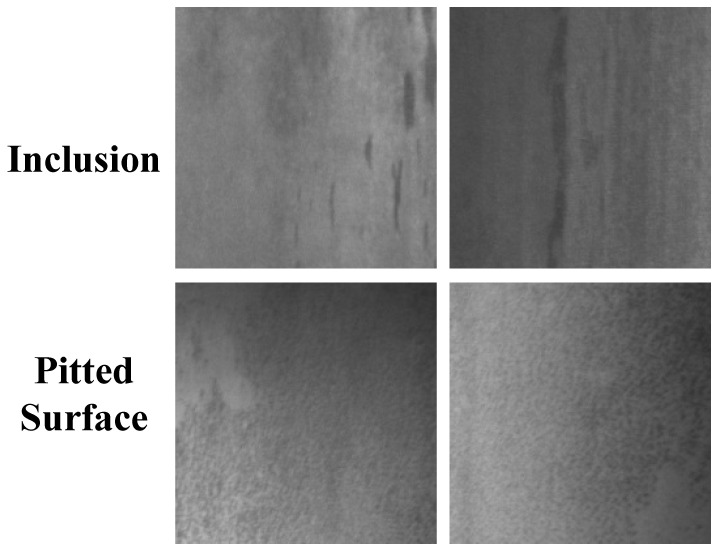
Case study. (Row 1): Inclusion misclassified as Pitted Surface. (Row 2): Pitted Surface misclassified as Inclusion.

However, it should be pointed out that the two defect categories, Pitted Surface and Rolled-in Scale, are small in size, weak in edge definition, and present as dark clumps in images, resulting in low visual separability. Constrained by the 8.2 MB model size limitation, the receptive field and channel capacity are restricted, making it difficult to capture sufficiently fine morphological differences, thus leading to frequent confusion between these two categories. As can be seen from Figure 11, both types of defects are mainly dark clumps with approximately circular or elliptical shapes and low edge contrast. When a pit has a small area, smooth edges, and internal oxidation shadows, its grayscale distribution almost overlaps with that of dark inclusions, forming the so-called “pseudo-homogeneous” feature, which makes it difficult for the model to distinguish subtle morphological differences.

Although MobileSteelNet achieves the highest overall accuracy, its performance varies across different defect categories due to dataset characteristics, model architectural limitations, and parameter sensitivities. We provide detailed explanations for cases where other models outperform MobileSteelNet.

Scratches Category (RepViT-M1: 99.45% vs. MobileSteelNet: 93.70%): Although MobileSteelNet showed dramatic improvement over the baseline (82.12 percentage points), the transformer-based RepViT-M1 performed slightly better on these elongated linear defects. This is likely due to the transformer architectures’ superior capability in modeling sequential patterns and directional dependencies, which are crucial for scratch-like features.

Inclusion Category (MobileNetV3: 96.49% vs. MobileSteelNet: 94.81%): This category consists of non-metallic impurities appearing as circular or irregular shapes with varying sizes. MobileNetV3’s inverted residual structure with squeeze-and-excitation blocks provided superior feature extraction for these blob-like defects, particularly under varying illumination conditions. MobileSteelNet’s performance was limited by its lightweight constraint (8.2 MB), which restricts the depth and width needed for optimal circular shape discrimination.

Patches Category (FastViT-SA12: 100.00% vs. MobileSteelNet: 93.70%): Patches are local irregular regions requiring strong spatial understanding. The transformer-based FastViT-SA12 excels at capturing long-range dependencies and irregular spatial patterns through self-attention mechanisms. MobileSteelNet’s CNN-based architecture, while efficient for multi-scale feature fusion, is less adept at modeling the complex spatial relationships inherent in irregular patch morphologies.

Pitted Surface Category (MobileOne-S0: 90.45% vs. MobileSteelNet: 68.27%): This represents the most significant performance gap. Pitted Surface defects appear as dark, shallow depressions with low contrast and weak edge definition. The MobileOne-S0 architecture, optimized for mobile deployment with reparameterization techniques, better handles these subtle low-contrast features. MobileSteelNet’s CIEMA module, while effective for most categories, struggles with these faint morphological variations due to the limited receptive field and channel capacity constraints imposed by the lightweight design.

Rolled-in Scale Category (Multiple models: 100.00% vs. MobileSteelNet: 98.15%): Rolled-in Scale defects are relatively distinct oxide layers with clear boundaries. Most models perform well on this category, with MobileSteelNet achieving near-perfect performance. The slight underperformance may be attributed to sensitivity to the specific regularization parameters used, as this category is less challenging and benefits more from simpler architectures.

Underlying Factors: These performance variations stem from three main factors: (1) Dataset Characteristics—the defects vary significantly in visual complexity, contrast, and morphological patterns; (2) Model Architecture Trade-offs—MobileSteelNet prioritizes efficiency over specialized architectural advantages of other models; and (3) Parameter Sensitivity—lightweight models are more sensitive to hyperparameter settings and data augmentation strategies.

#### 3.2.2. Comparison with Mainstream Attention Mechanisms

To verify the effectiveness and superiority of the proposed Cross-Interactive Efficient Multi-Scale Attention (CIEMA) mechanism, systematic comparative experiments with various mainstream attention mechanisms were conducted under the same MobileNetV1 backbone and identical experimental settings. These comparative methods include the following: Squeeze-and-Excitation (SE) [17], Convolutional Block Attention Module (CBAM) [19], Efficient Channel Attention (ECA) [18], coordinate attention (CA) [20], Efficient Multi-Scale Attention (EMA) [21]. All models were compared under the same experimental environment, training strategy, and dataset settings to ensure fairness of experiments. The experimental results are presented in Table 5.

The experimental results demonstrate the following: (1) Attention mechanisms that only focus on the channel dimension, such as SE and ECA, have limited effectiveness and even lead to performance degradation in some cases, indicating that using single-dimensional attention makes it difficult to meet the requirements of complex defect classification; (2) CBAM achieved satisfactory results (87.79%) by combining channel and spatial attention, but it still fell short of the proposed method; and (3) the CIEMA module achieved an average accuracy of 91.36%, surpassing all comparative attention mechanisms, thereby verifying its effectiveness in enhancing feature representation capability and model performance. Particularly noteworthy is that CIEMA achieved a recognition rate of 93.70% on the Scratches category, representing an improvement of 10.37 percentage points compared to the second-best CBAM (83.33%), fully demonstrating the importance of cross-spatial learning for elongated linear defect recognition.

A comprehensive comparison of the model performance based on Table 6 reveals distinct efficiency-performance trade-offs across different model categories. Heavyweight ResNet models (ResNet18-50) deliver robust accuracy (83.74–88.01%) but suffer from high computational costs (1.82–4.13 GFLOPs) and low inference speeds (14.36–50.09 FPS), making them unsuitable for real-time industrial applications. Lightweight MobileNet series shows better efficiency with MobileNetV2, achieving 86.08% accuracy at 0.30 GFLOPs and 42.11 FPS, while MobileNetV3 further optimizes to 0.22 GFLOPs. ShuffleNet models excel in speed with ShuffleNetV2 reaching 65.78 FPS, though at the cost of slightly lower accuracy (83.27%). Among the latest efficient architectures, EdgeNeXt-S provides a balanced profile with 84.72% accuracy and 54.38 FPS, while our MobileSteelNet achieves the optimal balance with 91.36% accuracy, 0.93 GFLOPs, and 52.04 FPS, outperforming all competitors in the accuracy-efficiency Pareto front. This analysis demonstrates MobileSteelNet’s superiority for industrial steel surface defect detection, where both high accuracy and real-time performance are critical requirements.

### 3.3. Ablation Experiments

#### 3.3.1. Module Effectiveness Validation

To further verify the effectiveness of the proposed multi-scale feature fusion (MSFF) module and Cross-Interactive Efficient Multi-Scale Attention (CIEMA) module, systematic ablation experiments were conducted on the NEU-DET dataset. MSFF and CIEMA modules were gradually introduced into the MobileNetV1 base network, and detection performance under different combinations was compared. The experiments include the following four configurations.

1. Baseline (MobileNetV1): The original MobileNetV1 network, serving as the baseline model;
2. MobileNetV1+MSFF: Introducing the multi-scale feature fusion (MSFF) module into the baseline network to integrate features at different scales;
3. MobileNetV1+CIEMA: Introducing only the proposed CIEMA attention module to enhance inter-channel and inter-spatial feature representation;
4. MobileSteelNet: Simultaneously introducing MSFF and CIEMA modules, which represents the final network structure.

The experimental results are presented in Table 7. The following conclusions can be drawn from the results.

(1) Contribution of the MSFF module: Without introducing any improvements, MobileNetV1 achieved an average accuracy of 79.52%, with limited overall performance. After introducing the multi-scale feature fusion (MSFF) module, the average accuracy improved to 86.00% (+6.48%), indicating that multi-scale feature fusion enables the model to capture detailed information of defects at different sizes. Particularly on the Scratches category, the recognition rate improved from 38.89% to 66.30% (+27.41%), demonstrating that multi-scale fusion is especially effective for recognizing elongated linear defects.

(2) Contribution of the CIEMA module: When only the CIEMA module was added, the model’s average accuracy significantly improved to 90.93% (+11.41%), indicating that this module exhibits significant effectiveness in enhancing inter-channel dependencies and spatial feature modeling. On the Patches category, it achieved the highest recognition rate of 97.78%, demonstrating that CIEMA’s cross-spatial learning mechanism can effectively capture features of irregularly shaped defects.

(3) Synergistic effect of the two modules: When MSFF and CIEMA modules were combined simultaneously, the model performance further improved to 91.36%, achieving optimal results on most categories. Particularly on the Scratches category, it reached 93.70%, representing an improvement of 54.81 percentage points compared to the baseline, thereby verifying the complementary and synergistic gain effects of the two modules.

As shown in Table 8, under the setting of 100 training images/100 test images, MobileSteelNet increased the training time by 29.2% compared with the baseline MobileNetV1, while the test time only increased by 0.19 ms. The FPS dropped from 57.9 to 52.0, but still maintained the real-time inference capability of >50 FPS. This proves that, while significantly improving accuracy, it has a controllable impact on computational efficiency and is suitable for edge deployment.

#### 3.3.2. Confusion Matrix Analysis

**Figure 12:**
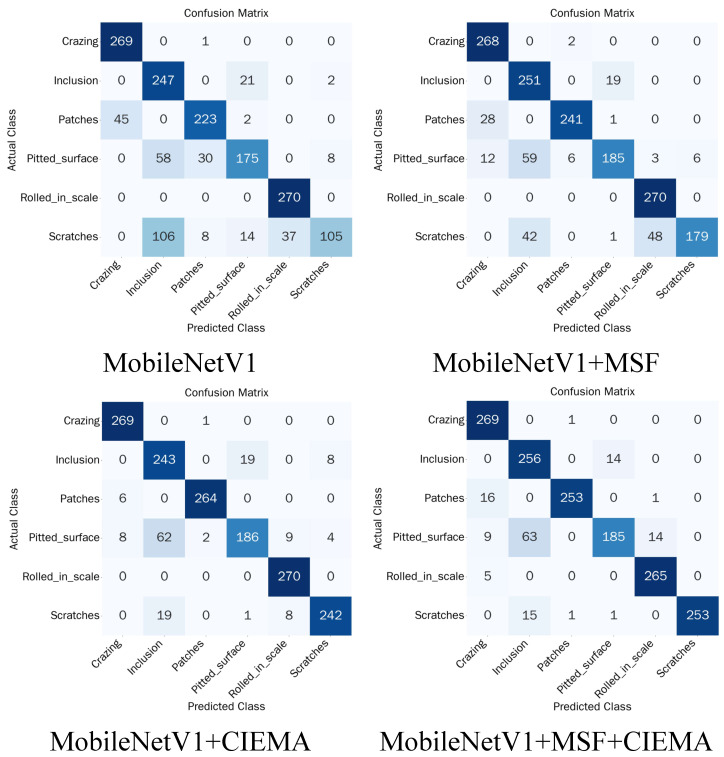
Ablation confusion matrix.

To more intuitively demonstrate the impact of different modules on the model’s classification discriminative capability, Figure 12 illustrates the confusion matrix comparison of ablation experiments.

The following patterns can be observed from the confusion matrices.

(1) The baseline model has weak recognition capability for Scratches category defects, with a 39.25% probability of misclassifying as Inclusion, 13.7% probability of misclassifying as Rolled-in Scale, and lower probabilities of misclassifying as Patches and Pitted Surface. After introducing MSFF and CIEMA modules separately on the baseline model, the misclassification rate of Scratches decreased from 61.11% to 33.7% and 10.37%, respectively. Finally, MobileSteelNet, formed by simultaneously adding MSFF and CIEMA modules to the baseline model, reduced the misclassification rate of Scratches significantly to 6.3%, fully demonstrating the effectiveness of MobileSteelNet improvements.

(2) The baseline model had a 16.67% probability of misclassifying Patches as Crazing, which decreased to 10.37% after adding the MSFF module and further decreased to 2.96% after adding the CIEMA module. However, after adding both modules, this misclassification rate slightly increased to 5.92%, indicating that the multi-scale information introduced by MSFF may cause slight interference with CIEMA’s fine-grained discrimination in some cases but that its overall performance is still significantly better than single-module configurations.

(3) The baseline model frequently misclassified Pitted Surface category defects as Inclusion and Patches. After separately introducing MSFF and CIEMA modules, the probability of misclassifying Pitted Surface as Patches decreased significantly. MobileSteelNet, formed by simultaneously adding MSFF and CIEMA modules to the baseline model, reduced the number of samples misclassified as Patches to 0, indicating that the model has stronger perception capability for fine-grained image classification. However, the confusion probability between Pitted Surface and Inclusion remains relatively large, indicating that further refinement is warranted in this aspect.

#### 3.3.3. CIEMA Internal Module Ablation

To verify the effectiveness of CIEMA attention improvements based on EMA, ablation experiments were conducted on the internal modules of CIEMA attention. As shown in Table 9, the experimental results indicate that both channel interaction and Cross Spatial Learning play important roles in steel surface defect classification.

From the experimental results, the following can be observed: (1) after removing the Cross Spatial Learning module, the average accuracy decreased to 89.88% (−1.48%), indicating that cross-spatial learning plays an important role in integrating multi-scale spatial information; (2) after removing the channel interaction module, the average accuracy significantly decreased to 85.63% (−5.73%), especially with respect to the Scratches category, which dropped sharply from 93.70% to 63.70%, demonstrating that the channel interaction mechanism is crucial for modeling inter-channel dependencies; and (3) the synergistic effect of the two modules enabled the complete CIEMA module to achieve optimal overall performance, validating the rationality of the design.

## 4. Discussion

Based on the aforementioned experimental results, this section provides an in-depth discussion of the proposed MobileSteelNet network, analyzing its advantages, comparing it with existing methods, and detailing its existing limitations. The discussion is organized into three subsections: the advantages and innovation of the method, comparative analysis with existing methods, and the limitations of the method and future work.

### 4.1. Advantages and Innovation of the Method

The proposed MobileSteelNet network achieves significant performance improvements in steel surface defect classification tasks by introducing the multi-scale feature fusion (MSFF) module and Cross-Interactive Efficient Multi-Scale Attention (CIEMA) module. Specifically, the advantages of this method are reflected in the following aspects.

First, effectively addressing complex defect classification challenges. In existing steel surface defect detection, complex defects, noise interference, and defect similarity are common challenges. Through multi-scale feature fusion (MSFF), this method can effectively integrate contextual information from different receptive fields, enhancing recognition capability for defects of different sizes. Particularly when handling small-scale defects, the MSFF module effectively avoids the loss of small defect information by aligning and fusing feature maps.

Second, enhancing fine-grained feature representation capability. The CIEMA module, through modeling inter-channel dependencies and introducing cross-spatial learning, provides the model with significant advantages in fine-grained spatial information modeling. Experiments demonstrate that this module can effectively improve classification performance, especially with respect to exhibiting stronger discriminative capability when defect categories are highly similar. Third, achieving a balance between accuracy and efficiency. Compared with traditional deep learning models (such as MobileNetV1 and ResNet), MobileSteelNet demonstrates significant advantages in both accuracy and computational efficiency. While maintaining low computational overhead, the model achieves superior performance in defect classification, making MobileSteelNet not only suitable for high-precision defect detection tasks, but also capable of meeting the real-time and efficiency requirements in industrial production.

### 4.2. Comparative Analysis with Existing Methods

This study conducted systematic comparative experiments with various mainstream methods, and the results demonstrate that the proposed method exhibits significant advantages.

At the network architecture level, MobileSteelNet surpasses heavy networks ResNet-50 (88.01%) and other lightweight networks, such as MobileNetV2 (86.08%) and ShuffleNetV1 (87.43%), with an average accuracy of 91.36%, thereby verifying that lightweight design and performance enhancement can be achieved simultaneously.

At the attention mechanism level, comparative experiments with attention mechanisms, such as SE, ECA, CBAM, coordinate attention, and EMA, demonstrate that the CIEMA module exhibits significant advantages in improving classification performance. Although the SE and ECA modules achieved satisfactory results in channel attention, they failed to fully utilize the fusion of spatial information and multi-scale information, thus performing weakly in complex defect classification. CBAM and EMA modules have improved by introducing spatial attention and multi-scale strategies, but they still have issues with high computational complexity. In contrast, the CIEMA module, through effective combination of channel interaction and cross-spatial learning, significantly improves the model’s classification accuracy while maintaining computational efficiency.

In terms of specific category performance, MobileSteelNet performs particularly prominently in categories such as Scratches and Patches. Through ablation experiments, the synergistic effects of the MSFF and CIEMA modules were verified, and their combination significantly improved the overall performance of the model, thereby demonstrating the complementarity and gain effects between modules.

### 4.3. Limitations of the Method and Future Work

Although MobileSteelNet achieved promising performance in multiple aspects, several limitations remain that warrant attention in future work.

(1) Although the MSFF module can effectively integrate multi-scale features, certain limitations may persist in detecting extremely small defects. Future work can explore more fine-grained feature extraction methods, such as introducing deformable convolution or dense connection structures, to further improve classification accuracy for small defects.

(2) The CIEMA module has increased computational complexity compared to traditional methods. Although the advantages were verified through ablation experiments, computational efficiency on large-scale industrial datasets warrants further optimization. Future work can consider further reducing model size and improving real-time performance through model pruning, knowledge distillation, or quantization techniques.

(3) From confusion matrix analysis, it can be observed that the confusion probability between Pitted Surface and Inclusion remains relatively large. These two types of defects exhibit certain similarities in visual features, requiring further optimization of feature representation or introduction of additional supervisory signals to enhance discriminative capability.

(4) Although this study primarily focuses on the classification of steel surface defects, the method exhibits good generality. Future work can attempt to apply this method to defect detection tasks for other materials, such as plastic surfaces and glass surfaces, to verify its cross-domain applicability.

## 5. Conclusions

This work addresses the problem of difficulty in balancing accuracy and efficiency in steel surface defect classification tasks, proposing a lightweight network MobileSteelNet based on MobileNetV1, combining the multi-scale feature fusion (MSFF) module and Cross-Interactive Efficient Multi-Scale Attention (CIEMA) module. The MSFF module effectively integrates multi-scale features from different network stages through bilinear interpolation and channel fusion, enhancing perception capability for defects of different sizes. The CIEMA module, for the first time within a lightweight framework, unifies explicit inter-channel interaction, multi-scale spatial feature extraction, and adaptive fusion, as well as groups efficient computation with global semantic modeling, thereby significantly enhancing feature discriminability. MobileSteelNet achieved a classification accuracy of 91.36% on the NEU-DET dataset, surpassing heavy networks, such as ResNet-50 (88.01%), and mainstream attention mechanisms, such as CBAM (87.79%). Particularly on the Scratches category, the recognition rate improved from the baseline of 38.89% to 93.70%, fully verifying the effectiveness of the proposed method. Furthermore, the model size was only 8.2 MB, demonstrating excellent lightweight characteristics suitable for deployment in industrial vision sensor systems and edge computing devices.

The proposed method still exhibits certain confusion in distinguishing between Pitted Surface and Inclusion. Future work can be improved in the following directions: (1) exploring more efficient model compression techniques to further reduce computational overhead; (2) introducing hard example mining or contrastive learning strategies to enhance discriminative capability for frequently confused categories; and (3) extending this method to surface defect detection for other materials to verify its cross-domain generality.

## Figures and Tables

**Table 1:** Input–output dimensions and the symbolic representation of each layer in the improved MobileNetV1 model.

Layer Name	Input Size	Output Size	Symbolic Representation
Input	(1, 3, 200, 200)	-	$X_{0}$
Init Layer	(1, 3, 200, 200)	(1, 32, 100, 100)	$X_{1}=\mathrm{ReLU}\left(\mathrm{BN}\left({\mathrm{Conv}}_{3x3,s=2,p=1}\left(X_{0}\right)\right)\right)$
Stage1 (0)	(1, 32, 100, 100)	(1, 64, 100, 100)	$X_{2}=\mathrm{ReLU}\left(\mathrm{BN}\left(\mathrm{DSConv}\left(X_{1}\right)\right)\right)$
Stage1 (1)	(1, 64, 100, 100)	(1, 128, 50, 50)	$X_{3}=\mathrm{ReLU}\left(\mathrm{BN}\left({\mathrm{DSConv}}_{s=2}\left(X_{2}\right)\right)\right)$
Stage1 (2)	(1, 128, 50, 50)	(1, 128, 50, 50)	$X_{4}=\mathrm{ReLU}\left(\mathrm{BN}\left(\mathrm{DSConv}\left(X_{3}\right)\right)\right)$
Stage2 (0)	(1, 128, 50, 50)	(1, 256, 50, 50)	$X_{5}=\mathrm{ReLU}\left(\mathrm{BN}\left(\mathrm{DSConv}\left(X_{4}\right)\right)\right)$
Stage2 (1)	(1, 256, 50, 50)	(1, 512, 25, 25)	$X_{6}=\mathrm{ReLU}\left(\mathrm{BN}\left({\mathrm{DSConv}}_{s=2}\left(X_{5}\right)\right)\right)$
Stage2 (2)	(1, 512, 25, 25)	(1, 512, 25, 25)	$X_{7}=\mathrm{ReLU}\left(\mathrm{BN}\left(\mathrm{DSConv}\left(X_{6}\right)\right)\right)$
Stage2 (3)	(1, 512, 25, 25)	(1, 512, 25, 25)	$X_{8}=\mathrm{ReLU}\left(\mathrm{BN}\left(\mathrm{DSConv}\left(X_{7}\right)\right)\right)$
Stage3 (0)	(1, 512, 25, 25)	(1, 512, 25, 25)	$X_{9}=\mathrm{ReLU}\left(\mathrm{BN}\left(\mathrm{DSConv}\left(X_{8}\right)\right)\right)$
Stage3 (1)	(1, 512, 25, 25)	(1, 512, 25, 25)	$X_{10}=\mathrm{ReLU}\left(\mathrm{BN}\left(\mathrm{DSConv}\left(X_{9}\right)\right)\right)$
Stage3 (2)	(1, 512, 25, 25)	(1, 1024, 13, 13)	$X_{11}=\mathrm{ReLU}\left(\mathrm{BN}\left({\mathrm{DSConv}}_{s=2}\left(X_{10}\right)\right)\right)$
Stage3 (3)	(1, 1024, 13, 13)	(1, 1024, 13, 13)	$X_{12}=\mathrm{ReLU}\left(\mathrm{BN}\left(\mathrm{DSConv}\left(X_{11}\right)\right)\right)$
MSFF	(1, 1024, 50, 50)	(1, 1024, 50, 50)	$X_{13}=\mathrm{MSFF}\left(X_{4},X_{8},X_{12}\right)$
CIEMA	(1, 1024, 50, 50)	(1, 1024, 50, 50)	$X_{14}=\mathrm{CIEMA}\left(X_{13}\right)$
Global Avg Pool	(1, 1024, 50, 50)	(1, 1024, 1, 1)	$X_{15}=\mathrm{GAP}\left(X_{14}\right)$
Linear Layer	(1, 1024, 1, 1)	(1, 6)	$\hat{Y}=WX_{15}+b$

**Table 2:** Characteristics of steel surface defects in the NEU-DET dataset.

Defect Type	Defect Characteristics
Crazing	Fine cracks, usually appearing as irregular textures or stripes on the surface, typically appearing shallow with complex morphology.
Inclusion	Non-metallic impurities appearing on the steel surface, usually manifested as circular or irregular shapes with varying sizes.
Patches	Local patch-like defects on the surface, with irregular morphology, usually appearing as spot-like or irregular regions.
Pitted Surface	Surface showing relatively deep pits with uneven spacing and large depth, usually affecting the flatness of steel.
Rolled-in Scale	Surface with oxide scale attached during the rolling process, with the oxide layer showing irregular shapes.
Scratches	Linear or strip-like scratches on the surface, usually caused by external forces, with varying depth and length.

**Table 3:** Contrast statistics for each category.

Category	Max	Min	Median
Crazing	51.5440	18.9998	28.5290
Inclusion	36.5146	5.9717	11.2242
Patches	74.2065	17.5567	57.0919
Pitted	47.5536	10.3722	26.3910
Rolled-in Scale	27.4891	8.9324	15.9555
Scratches	49.0211	7.3541	20.3314

**Table 4:** Comparative experimental results of the different models on the NEU-DET dataset. The bold values in the table indicate the maximum values.

Model	Avg Acc	Crazing	Inclusion	Patches	Pitted Surface	Rolled-In Scale	Scratches
ResNet18 [11]	83.74%	98.25%	91.58%	77.54%	58.95%	99.65%	76.49%
ResNet34 [11]	81.23%	97.54%	88.07%	64.56%	64.91%	99.30%	72.98%
ResNet50 [11]	88.01%	97.19%	91.23%	89.82%	64.91%	**100.00%**	84.91%
MobileNetV1 [14]	70.29%	94.39%	90.88%	75.79%	58.60%	90.53%	11.58%
MobileNetV2 [15]	86.08%	97.54%	93.68%	85.26%	62.11%	96.14%	81.75%
MobileNetV3 [22]	78.89%	97.19%	**96.49%**	70.88%	58.25%	97.89%	52.63%
ShuffleNetV1 [23]	87.43%	97.89%	93.68%	91.58%	70.18%	**100.00%**	71.23%
ShuffleNetV2 [24]	83.27%	96.84%	93.68%	89.12%	60.70%	98.60%	60.70%
MobileOne-S0 [25]	88.13%	83.75%	69.12%	99.53%	**90.45%**	93.03%	92.91%
EdgeNeXt-S [26]	84.72%	83.18%	83.33%	98.74%	84.44%	87.59%	71.02%
RepViT-M1 [27]	73.19%	66.18%	56.25%	89.11%	87.62%	40.53%	**99.45%**
FastViT-SA12 [28]	74.47%	48.60%	70.76%	**100.00%**	70.89%	78.12%	78.44%
Ours	**91.36%**	**99.63%**	94.81%	93.70%	68.27%	98.15%	93.70%

**Table 5:** Comparative experimental results of the different attention mechanisms on the NEU-DET dataset. The bold values in the table indicate the maximum values.

Model	Avg Acc	Crazing	Inclusion	Patches	Pitted Surface	Rolled-In Scale	Scratches
+MSFF	86.00%	99.26%	92.96%	89.26%	68.27%	**100.00%**	66.30%
+MSFF+SE	79.77%	98.89%	92.22%	81.11%	67.53%	94.81%	44.07%
+MSFF+ECA	80.51%	99.26%	91.48%	71.11%	70.11%	**100.00%**	51.11%
+MSFF+CBAM	87.79%	**100.00%**	91.85%	85.93%	70.85%	94.81%	83.33%
+MSFF+CoordAtt	84.70%	99.26%	92.96%	85.93%	**71.22%**	**100.00%**	58.89%
+MSFF+EMA	86.06%	98.52%	91.11%	**86.67%**	68.63%	**100.00%**	71.48%
+MSFF+CIEMA	**91.36%**	99.63%	**94.81%**	93.70%	68.27%	98.15%	**93.70%**

**Table 6:** Model parameter counts, GFLOPs, and FPS.

Model	Total Parameter Count	GFLOPS	FPS
ResNet18 [11]	11,689,512	1.82	50.09
ResNet34 [11]	21,797,672	3.68	25.19
ResNet50 [11]	25,557,032	4.13	14.36
MobileNetV1 [14]	4,231,976	0.57	57.90
MobileNetV2 [15]	3,504,872	0.30	42.11
MobileNetV3 [22]	5,483,032	0.22	49.43
ShuffleNetV1 [23]	1,531,936	0.15	68.55
ShuffleNetV2 [24]	2,278,604	0.15	65.78
MobileOne-S0 [25]	5,293,272	1.05	38.56
EdgeNeXt-S [26]	5,586,832	0.96	54.38
RepViT-M1 [27]	5,485,248	0.85	37.59
FastViT-SA12 [28]	11,580,968	1.49	35.72
Ours	7,068,679	0.93	52.04

**Table 7:** Ablation experimental results on the NEU-DET dataset. The bold values in the table indicate the maximum values.

Model	Avg Acc	Crazing	Inclusion	Patches	Pitted Surface	Rolled-in Scale	Scratches
Baseline	79.52%	99.63%	91.48%	82.59%	64.58%	**100.00%**	38.89%
Baseline+MSFF	86.00%	99.26%	92.96%	89.26%	68.27%	**100.00%**	66.30%
Baseline+CIEMA	90.93%	99.63%	90.00%	**97.78%**	**68.63%**	**100.00%**	89.63%
Ours	**91.36%**	**99.63%**	**94.81%**	93.70%	68.27%	98.15%	**93.70%**

**Table 8:** Efficiency ablation study on 100 training images and 100 test images.

Model	Training Time (s)	Testing Time (s)	FPS
Baseline	1440.05	1.73	57.90
Baseline+MSFF	1590.30	1.79	56.02
Baseline+CIEMA	1692.47	1.82	54.83
Ours	1860.88	1.92	52.04

**Table 9:** Ablation experiments on CIEMA attention internal modules.The bold values in the table indicate the maximum values.

Model	Avg Acc	Crazing	Inclusion	Patches	Pitted Surface	Rolled-in Scale	Scratches
w/o CrossSpatialLearning	89.88%	**99.63%**	91.11%	89.26%	**70.85%**	93.33%	**95.19%**
w/o ChannelInteraction	85.63%	98.52%	92.59%	88.89%	70.11%	**100.00%**	63.70%
Ours	**91.36%**	**99.63%**	**94.81%**	**93.70%**	68.27%	98.15%	93.70%

## Data Availability

The data presented in this study are openly available in the NEU surface defect database at https://drive.google.com/file/d/1qrdZlaDi272eA79b0uCwwqPrm2Q_WI3k/view (accessed on 13 August 2025).

## Abbreviations

The following abbreviations are used in this manuscript:

MSFF	Multi-Scale Feature Fusion
CIEMA	Cross-Interactive Efficient Multi-Scale Attention
DSConv	Depthwise Separable Convolution
GAP	Global Average Pooling
BN	Batch Normalization
SE	Squeeze-and-Excitation
CBAM	Convolutional Block Attention Module
ECA	Efficient Channel Attention
CA	Coordinate Attention
EMA	Efficient Multi-Scale Attention
CNN	Convolutional Neural Network
FPN	Feature Pyramid Network
